# Electronic-Structure Role of Cobalt Oxide Nanoparticles in P3HT-Based Composites: From Thin-Film Fabrication to DFT-Assisted Surface Interaction Analysis

**DOI:** 10.1021/acsomega.6c00453

**Published:** 2026-04-22

**Authors:** Diego Hernández-Martínez, Luis E. López-González, Sarahi García-Carvajal, Aurora M. Pat-Espadas, Karla A. López-Gastelum, Damian F. Plascencia-Martínez, Guillermo Suárez-Campos, María E. Nicho-Díaz

**Affiliations:** † Departamento de Ingeniería Química y Metalurgia, Universidad de Sonora, Del Conocimiento, Centro, Hermosillo, Sonora C.P. 83000, México; ‡ Centro de Nanociencias y Nanotecnología, Universidad Nacional Autónoma de México, Km 107 Carretera Tijuana-Ensenada S/N, Ensenada, Baja California C.P. 22800 México; § Escuela Nacional de Estudios Superiores Unidad León, Universidad Nacional Autónoma de México, Boulevard UNAM #2011 Col. Predio El Saucillo y El Potrero Comunidad de Los Tepetates, León, Guanajuato C.P. 37684, México; ∥ Departamento de Investigación y Posgrado en Alimentos, Universidad de Sonora, Rosales y Blvd. Luis Encinas, Hermosillo, Sonora C.P. 83000, México; ⊥ Coordinación de Tecnología de Alimentos de Origen Vegetal, Centro de Investigación en Alimentación y Desarrollo, A.C. Carr. Gustavo E. Astiazarán Rosas No. 46, Col. La Victoria, Hermosillo, Sonora C.P. 83304, Mexico; # Departamento de Investigación en Física, Universidad de Sonora, Blvd Luis Encinas y Rosales S/N, Hermosillo, Sonora C.P. 83000 México; ∇ Centro de Investigación en ingeniería y Ciencias Aplicadas, Universidad Autónoma del Estado de Morelos, Av. Universidad 1001, Col. Chamilpa, Cuernavaca, Morelos C.P. 62209, México

## Abstract

The present work aims to obtain composites of two semiconductor materials, poly­(3-hexylthiophene) (P3HT) and cobalt oxide (Co_3_O_4_), by in situ synthesis using the Sugimoto method. Both materials serve as hole-transport layers (HTL) in photovoltaic devices. The objective is to understand the effect of Co_3_O_4_ nanoparticles on P3HT, particularly how they interact and potentially alter the polymer’s electronic structure. Co_3_O_4_ nanoparticles were synthesized by chemical precipitation as a byproduct of thin-film formation. They were incorporated at different concentration weight ratios (P3HT/Co_3_O_4_:1/0.03, 1/0.05, and 1/0.08) into the P3HT/Co_3_O_4_ composite. Their influence on the optoelectronic properties was analyzed. P3HT/Co_3_O_4_ films were characterized by various techniques: morphological (SEM, TEM, and AFM) and optoelectronic (FTIR and photoluminescence). The objective of the resulting composite is to improve HTL performance in a solar cell. Additionally, a DFT+U computational study was performed to investigate the P3HT adsorption on the Co_3_O_4_(110) surface. Three configurations showed that Co–S bond formation and ligand-to-metal charge transfer are the primary interactions, with van der Waals forces contributing significantly to the adsorption energy. Overall, the effect of cobalt oxide on P3HT was investigated experimentally and theoretically, contributing to a deeper understanding of the interfacial electronic interactions in P3HT/Co_3_O_4_ systems beyond conventional structural and optoelectronic analyses.

## Graphical abstract

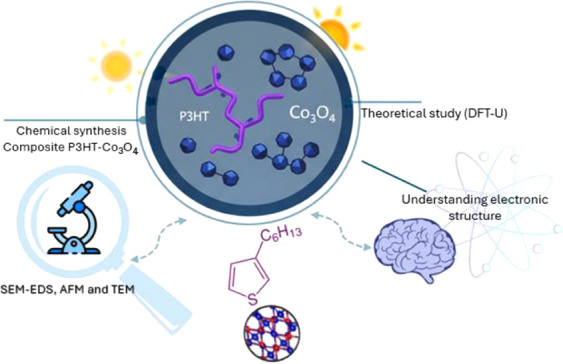

## 1 Introduction

Several materials have been explored as hole transport layers (HTLs) in bulk heterojunction (BHJ) solar cells, including conducting polymers,^1^ conjugated polyelectrolytes (CPEs)^2^ metal oxides and graphene-based materials. An efficient HTL must behave as a wide-bandgap p-type semiconductor,^3^ enabling selective hole extraction while blocking electrons and improving device stability. Among inorganic HTLs, WO_3_, CuO, Sb_2_O_3_, V_2_O_5_, and MoO_3_, have been reported with NiO being the most effective, with efficiencies exceeding 6%.^4^

In parallel, organic HTLs such as regioregular and regiorandom poly­(3-hexylthiophene) (P3HT), poly­(2,6-(4,4-bis­(2-ethylhexyl)-4*H*-cyclopenta­[2,1-*b*;3,4-*b*′]­dithiophene)-*alt*-4,7­(2,1,3-benzothiadiazole)) (PCPDTBT), and poly­(triarylamine) (PTAA) have demonstrated excellent processability and tunable optoelectronic properties. Among them, P3HT remains one of the most extensively studied poly­(3-alkylthiophene) (P3AT) systems due to its environmental stability, relatively high hole mobility, and solution-processable synthesis via oxidative polymerization (Sugimoto R^24^). Compared with PPV-based systems, P3HT exhibits improved absorption and charge-transport properties.

Interfacial interactions play a decisive role in determining polymer crystallinity and charge-transport mechanisms in hybrid systems, often favoring hopping and tunneling processes.^5^ For example, inorganic NiO_*x*_ layers have successfully acted as HTLs in organic solar cells (OSCs), yielding satisfactory device performance.^6^

Cobalt oxides CoO_*x*_ have attracted increasing attention not only as oxidation catalysts,^5,7^ and solar-selective absorbers, but also as promising HTL materials. Co_3_O_4_ has been proposed as a substitute for PEDOT:PSS in OSCs, achieving a power conversion efficiency (PCE) of 14.13% with improved stability using the architecture ITO/HTL (PEDOT:PSS or Co_3_O_4_).^8^ A PCE of 3.21% was also reported in ITO/PEDOT:PSS or Co_3_O_4_/PCDTBT:PC_71_BM/Al devices.^9^ These improvements are attributed to the high transparency (≈81%) and low roughness of Co_3_O_4_ layers, which enhance the fill factor and overall PCE.^4^

Similarly, perovskite solar cells (PSCs) incorporating CoO_*x*_ as HTL have demonstrated promising performance. A PCE of 14.5% was achieved in ITO/CoO_*x*_/CH_3_NH_3_PbI_3_/PCBM/Ag devices,^10^ while Co_3_O_4_ nanocrystal-based HTLs enabled a PCE of 8.92% in FTO/c-TiO_2_/m-TiO_2_/CsPbBr_3_/Co_3_O_4_/carbon architectures,^11^ highlighting their stability and compatibility with diverse photovoltaic platforms.

Beyond their standalone role as HTLs, Co_3_O_4_ nanoparticles have been incorporated into conjugated polymers to tailor interfacial and charge-transport properties. Ozkazanc E reported reduced trap dispersion and improved hole transport in polythiophene (PTh)/Co_3_O_4_ nanocomposites.^12^ Likewise, Yousaf et al. showed that adding Co_3_O_4_ nanoparticles to P3HT:PCBM active layers increases film roughness and promotes interpenetrating networks that facilitate charge transport.^13^ Additionally, the design of hybrid nanocomposites based on cobalt oxide grafted onto carbon quantum dots (Co_3_O_4_/CQDs) has demonstrated outstanding electrochemical performance in asymmetric supercapacitors, exhibiting high specific capacitance, excellent cyclic stability, and efficient charge transfer.^14^

More broadly, hybrid nanocomposites combining conducting polymers and metal oxides have attracted considerable interest for photocatalytic, sensing, and energy-storage applications. Preparation strategies include direct blending, sol–gel processing, and in situ polymerization.^15^ Examples include PANI–SnO_2_, PPy–SnO_2_, PEDT–SnO_2_, and P3HT–SnO_2_ systems for gas sensing,^16,17^ as well as PP/magnetite/MWCNT systems showing enhanced electromagnetic properties through multicomponent engineering.^18,19^ In situ approaches have also enabled PPy/graphite oxide composites and MoO_3_/PANI heterostructures with improved electrochemical performance.^20^

Importantly, several studies indicate that the sulfur atom of the thiophene ring plays a crucial role in interfacial interactions with metal oxides. For example, P3HT–FeO­(OH) systems exhibited bandgap modulation attributed to Fe–S interactions.^21^ Zhang et al. reported that P3HT interacts with TiO_2_ primarily through coordination between the thiophene sulfur and surface oxide sites, facilitating charge transfer.^22,23^ Although these studies support the existence of S–metal interactions, the electronic-structure implications of such bonding remain insufficiently understood, particularly for Co_3_O_4_-based systems.

Therefore, in this work, we combine experimental thin-film fabrication with DFT+U computational analysis to elucidate the interfacial interaction between P3HT and Co_3_O_4_ nanoparticles. By correlating morphological, optoelectronic, and theoretical results, we aim to clarify the role of Co–S bond formation, ligand-to-metal charge transfer, and van der Waals contributions in modulating the electronic structure of P3HT/Co_3_O_4_ composites. This integrated approach provides deeper insight into the interfacial electronic mechanisms governing hybrid HTLs, moving beyond conventional structural characterization toward a more comprehensive understanding of structure–property relationships.

## 2 Experimental Section

### 2.1 Materials

3-Hexylthiophene (C_10_H_16_S, 99%) and Iron Chloride (FeCl_3_, 97%) were provided by Sigma-Aldrich; Chloroform 99.8% purity (CHCl_3_) by Sigma-Aldrich, Methanol (CH_3_OH, 99.9%, Sigma-Aldrich), Acetone (CH_3_COCH_3_, 99.7%) Baker and Hexane 99% (C_6_H_14_), were provided by Fermont. 3-Hexylthiophene was distilled under reduced pressure and stored in the dark before use.

### 2.2 Synthesis of Co_3_O_4_

Co­(OH)_2_ powder was synthesized by chemical bath deposition. In this process, a 0.1 M cobalt sulfate solution (CoSO_4_·6H_2_O, 99% purity (Sigma-Aldrich)) was used as a source of Co^2+^ ions, and 3.7 M triethanolamine (Fermont), 99.8% purity (C_6_H_15_NO_3_), as a complexing agent, with a final volume of 100 mL. The reaction temperature was raised to 65 °C (pH ∼ 10), and the reaction was allowed to proceed for 1 h. The precipitates obtained from the homogeneous precipitation of Co­(OH)_2_ were recovered and washed with water and isopropanol (Sigma-Aldrich, 99.5% purity) by centrifugation at 6000 rpm for 15 min. Finally, the obtained Co_3_O_4_ powder was annealed at 400 °C for 2 h.

### 2.3 Synthesis of P3HT/Co_3_O_4_ Composites

For P3HT-Co_3_O_4_ composites, the 3-hexylthiophene monomer was polymerized by chemical oxidation (Sugimoto R^24^) using FeCl_3_ as the oxidant at 0 °C for 24 h under an inert atmosphere. The composites were synthesized with different weight ratios: 3HT/Co_3_O_4_ = 1/0.03, 1/0.05, and 1/0.08. The Co_3_O_4_ particles were first dispersed by sonication in CHCl_3_ and then added under magnetic stirring to the FeCl_3_ solution in CHCl_3_. The previously distilled 3HT monomer was dissolved in CHCl_3_ and carefully added to the Co_3_O_4_/FeCl_3_ solution. The mixture was stirred for 24 h at 0 °C. The product precipitated in methanol, filtered, and washed with methanol and hexane by centrifugation (10 min, 7000 rpm), and finally dried. For comparison, P3HT was synthesized without Co_3_O_4_ following the procedure described above. The yields for P3HT and composites were 79.55, 78.09, 76.41, and 66.74%, respectively. Thin films of P3HT and P3HT/Co_3_O_4_ composites were prepared on Corning glass substrates by spin-coating at a rotational speed of 2000 rpm for 120 s. Then, these films were subjected to thermal annealing for 10 min at 140 °C on a hot plate (all samples remained thermally stable up to 400 °C, determined by TGA), see Figure S4 for further details.

### 2.4 Computational Methods

Calculations were performed using the periodic density functional theory (DFT) framework as implemented in the PWscf code of the Quantum Espresso package.^25^ Exchange and correlation energies were treated using the generalized gradient approximation (GGA) with the Perdew, Burke, and Ernzerhof (PBE)^26^ gradient corrected functional, employing the Hubbard U correction scheme, with an effective Hubbard parameter (U_eff_ = U – J) of 3.30 eV applied.^27–29^ van der Waals interactions were considered using the Grimme DFT-D3 dispersion correction.^30,31^ A full optimization of the Co_3_O_4_ bulk structure was performed, as shown in Figure S1. Considering the bulk-optimized lattice parameters, we constructed a surface model employing the supercell method. We focused on the Co_3_O_4_(110) surface, as it is among the most stable Co_3_O_4_ surfaces.^32^ It was modeled using an eight-layer, periodically repeated slab geometry with a p(1 × √2) unit cell, with an 18 Å vacuum region between slabs to avoid image interactions. The supercell has dimensions of 8.000 × 11.314 × 26.314 Å.

The optimized structure of P3HT was determined from a two-ring thiophene model using a 7.989 × 20.000 × 20.000 Å unit cell at the Gamma k-point, considering the periodicity of the polymer chain in one direction.

The molecule–surface adsorption is analyzed using the following equation:

$$E_{\mathrm{ads}}=E_{\mathrm{mol}-\mathrm{slab}}-\left(E_{\mathrm{slab}}+E_{\mathrm{mol}}\right)$$

Where E_mol_, E_slab_, and E_mol–slab_ are the total energies of the isolated molecule, slab, and molecule slab systems.^33,34^ When chemisorption occurred, the character of the bonds was analyzed using Bader’s charge analysis.^35^ To understand the long-range interactions occurring during molecular surface adsorption, we have used the noncovalent interactions (NCI) index, as implemented in the CRITIC2 ^36^ code (extending the NCI method (Johnson et al.^36^) to solid-state periodic electron densities). Such analysis helps to reveal weak van der Waals (vdW) interactions, hydrogen bonding, and even repulsive interactions.

## 3 Characterization

To confirm the presence of all components in the systems spectroscopic evaluation was performed. To obtain the infrared (IR) spectra by attenuated total reflectance (ATR) in the range of 4000–400 cm^–1^ (PerkinElmer Frontier spectrometer). Fluorescence spectroscopy (PerkinElmer model LS50) was used with a 460 nm excitation source. ^1^H NMR spectra of P3HT and P3HT-Co_3_O_4_ composites were obtained on a Bruker Avance 400 MHz NMR, with tetramethylsilane (TMS) as the internal standard and CDCl_3_ as the solvent. Surface morphology was examined by TEM (JEOL 2010 F, Peabody (Massachusetts, USA)), JEOL model 5410LV (SEM), and AFM model Alpha300RA (WiTec, Germany).

## 4 Results and Discussion

### 4.1 FTIR-ATR Analysis

FTIR-ATR spectra (Figure 1) showed the characteristic bands of the P3HT polymer: 722 cm^–1^ rocking vibration of methylene groups of the hexyl −(CH_2_)_5_–, 820 cm^–1^ out-of-plane aromatic C–H vibration, 1115 cm^–1^ C–S stretching, the peaks at 1510 and 1455 cm^–1^ corresponds to thiophene ring C=C stretching vibration, asymmetric and symmetric, respectively, 1377 cm^–1^ is due to the methyl deformation, three bands at 2949, 2920, and 2852 cm^–1^ assigned to asymmetric −CH_3_ stretching vibrations, in-phase −CH_2_– vibrations and out-of-phase −CH_2_– vibrations, respectively.^37,38^ FTIR-ATR analysis shows a slight shift in the band from 820 to 822 cm^–1^ (Figure 2), this small change suggests that some nearby atom or group, possibly from the metal oxide, is approaching the P3HT ring. Since the motion of hydrogen in this vibration occurs outside the plane of the ring, the presence of this atom limits its motion, making it more rigid.^39,40^ This effect indicates that the polymer interacts with the oxide, likely via π–π interactions or interactions between sulfur and the metal.

**Figure 1:**
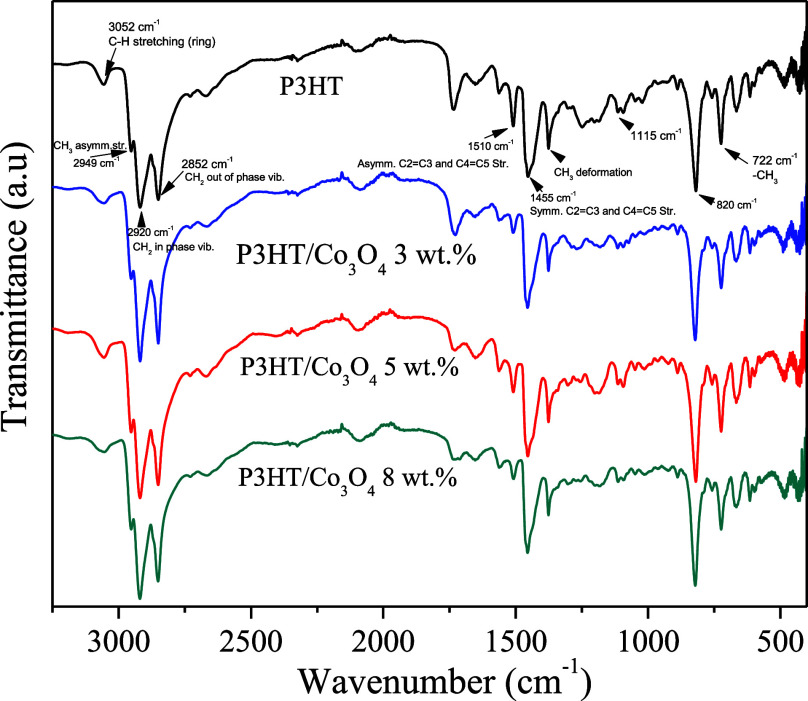
FTIR spectra of P3HT and P3HT/Co_3_O_4_ (3, 5, and 8 wt %).

**Figure 2:**
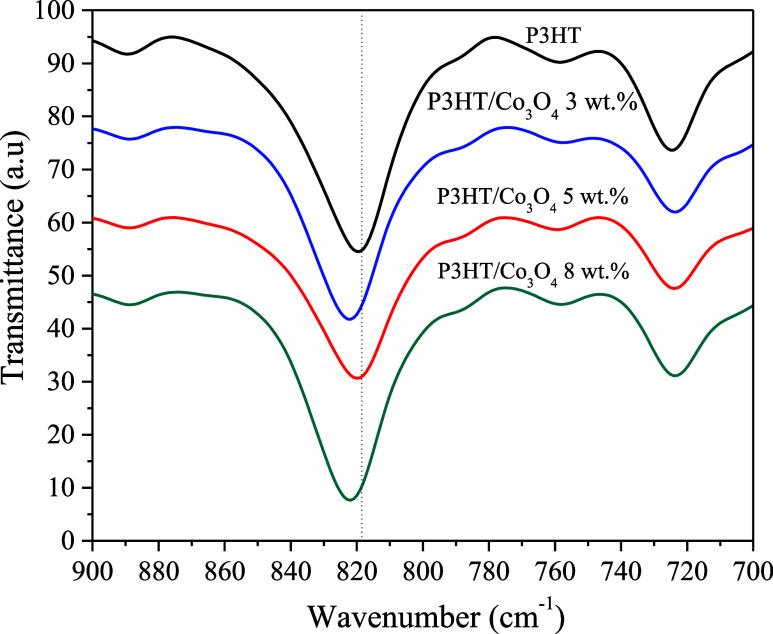
FTIR spectrum of the shift at 820 cm^–1^.

In addition, the broadening and slight decrease in the intensity of the 1115 cm^–1^ band (C–S stretching) were determined by comparing the full width at half-maximum (FWHM) and normalized absorbance of the P3HT and P3HT/Co_3_O_4_ spectra.^41^ This behavior is associated with a stronger interaction between sulfur and the metal oxide, which could be due to partial charge transfer or direct S–metal coordination. Together, these spectral changes reflect a good interaction between the polymer and the oxide nanoparticles, which favors a more efficient electronic connection.

According to Chen et al., the ratio of the intensities of symmetric C=C stretching (1455 cm^–1^) to that of asymmetric C=C stretching (1510 cm^–1^) indicates the conjugation length of the polymer chain.^42^ For longer conjugation lengths in the polymer chain, the I_sim_/I_asim_ ratio is smaller, and the longer the conjugation length, the higher the conductivity of the polymer chain. Table 1 shows the conjugation length calculations from the FTIR-ATR spectra for P3HT and P3HT/Co_3_O_4_ composites.

**Table 1:** Conjugation Lengths of P3HT and P3HT/Co_3_O_4_ Composites

Sample	Conjugation length (I_sim_/I_asim_)
P3HT	1.85
P3HT/Co_3_O_4_ (3 wt %)	4.05
P3HT/Co_3_O_4_ (5 wt %)	3.06
P3HT/Co_3_O_4_ (8 wt %)	4.33

The FTIR analysis indicates that increasing Co_3_O_4_ concentration does not modify the chemical structure of P3HT but progressively influences its molecular organization. At 3 wt %, slight shifts of the 820 cm^–1^ band and mild broadening of the 1115 cm^–1^ (C–S) vibration suggest the onset of S–Co interaction and subtle conformational rearrangement. At 5 wt %, these effects become more pronounced, indicating stronger interfacial coupling and modulation of the effective conjugation length. At 8 wt %, further C–S band broadening reflects enhanced S–Co coordination and increased conformational constraint near the oxide surface. Overall, the 3–5 wt % range provides the most favorable balance between interfacial interaction and preservation of polymer organization.

### 4.2 Photoluminescence Analysis

Figure 3 displays the photoluminescence (PL) spectra of P3HT/Co_3_O_4_ (3, 5 and 8 wt %). In the case of P3HT, the central emission peak is centered at 659 nm, which is typically related to electron–hole recombination. The peak’s low intensity could indicate reduced radiative recombination, possibly due to improved charge separation or enhanced light absorption.^43^ Its broad shape also suggests some structural disorder, which could hinder charge mobility and conductivity.^44^ Upon the addition of cobalt oxide, the emission peak becomes sharper and more intense. This change suggests improved molecular alignment and stronger interactions between P3HT and Co_3_O_4_. The slight blue shift observed at 3 wt % (from 659 to 649 nm) typically indicates a reduction in effective conjugation length and a decrease in the degree of π–π stacking, suggesting fewer exciton aggregates. This implies that a low nanoparticle concentration promotes chain dispersion and minimizes the formation of strongly coupled domains. At intermediate concentrations, nanoparticles enhance local radiative pathways. Research on Co_3_O_4_ and nanocrystalline films indicates that defects, crystallite size, and surface-state density significantly affect photoluminescence (PL).^45^ Additionally, the PL data reveal an optimal concentration range (approximately 3–5 wt %) where the P3HT/Co_3_O_4_ interface enhances local radiative processes, leading to the formation of emissive centers. Conversely, at higher concentrations, quenching effects and charge separation become dominant.^46^

**Figure 3:**
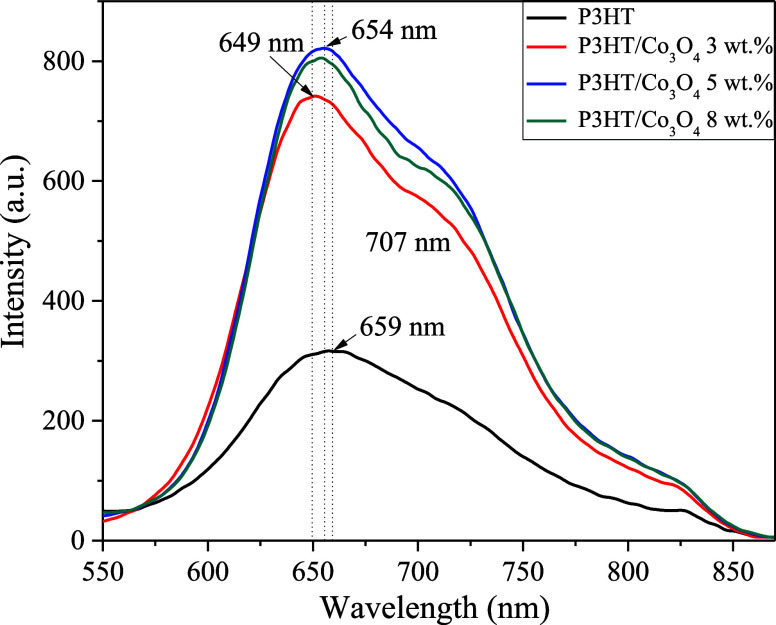
Photoluminescence (PL) spectra of P3HT and P3HT/Co_3_O_4_ (3, 5 and 8 wt %) with 460 nm of excitation.

The PL results show a nonlinear quenching behavior with respect to Co_3_O_4_ concentration: the 3 wt % composite exhibits the strongest quenching, followed by 8 wt %, whereas 5 wt % shows the weakest quenching among the composites. This suggests that quenching does not depend solely on the Co_3_O_4_ concentration, but rather on the balance between effective/accessible interfacial area, dispersion versus aggregation, and the possible formation of connected (percolation-like) pathways. At low loading (3 wt %), improved dispersion, with fewer aggregates, can maximize the number of accessible P3HT/Co_3_O_4_ interfacial sites, thereby promoting charge-transfer (or interfacial trapping) channels and reducing radiative recombination. At intermediate loading (5 wt %), more pronounced aggregation can reduce the effective interfacial area, thereby weakening quenching. At higher loading (8 wt %), although aggregates are more abundant, their improved distribution and more connected morphology (as suggested by AFM) can again increase the likelihood of charge-transfer pathways, resulting in stronger quenching than at 5 wt %. Since no devices were fabricated, these implications are discussed at the materials level; nevertheless, inorganic oxides (MoO_*x*_, NiO_*x*_, CoO_*x*_) have been reported to stabilize interfaces and contribute to operational stability^47,48^ and Co_3_O_4_ additives in P3HT:PCBM-type layers have been explored to enhance device performance.^13^

### 4.3 ^1^H NMR Analysis

The properties in function of the molecular arrangement of P3HT and its composites are essential to their overall properties; therefore, the head-to-tail (HT) regioregularity of all P3HT/Co_3_O_4_ composites was determined by NMR spectroscopy. Figure 4 shows the ^1^H NMR spectra of P3HT and P3HT/Co_3_O_4_ composites, the signal at 7.2 ppm corresponds to deuterated chloroform, the signals between 2.5–3.0 ppm correspond to CH_2_α, which is directly connected to the thiophene ring and reveals the dyad configuration, the signal at 2.8 ppm corresponds to the head–tail dyad (HT), and the signal at 2.55 ppm corresponds to the head–head dyad (HH). The signals between 6.9 and 7.1 ppm correspond to β-hydrogens (or hydrogens in 4-position) in the thiophene ring and reveal the triad configuration, for both the P3HT and P3HT/Co_3_O_4_ composites.^49,50^

**Figure 4:**
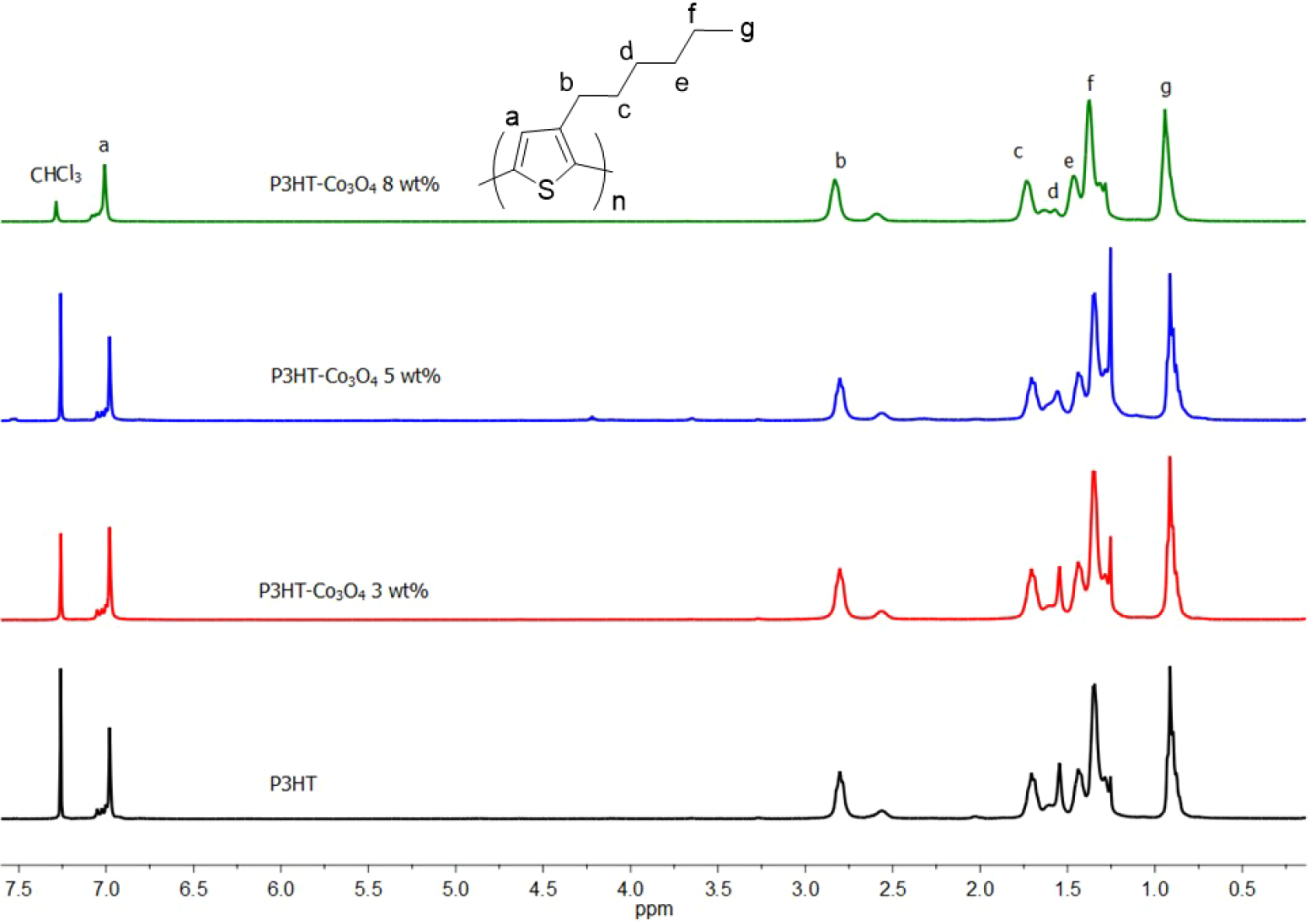
^1^H NMR spectrum of P3HT synthesized under different Co_3_O_4_ loading levels.

The contents of the head-to-head (HH) and head-to-tail (HT) dyads and the HT–HT, HT–HH, TT–HT, and TT–HH triads were estimated from the area under the curve of the signals that appeared around d = 2.58 ppm (HH), 2.80 ppm (HT), 6.98 ppm (HT–HT), 7.00 ppm (HT–HH), 7.03 ppm (TT–HT), and 7.05 ppm (TT–HH) in the ^1^H NMR spectrum; the results are presented in Table 2.^51^ The variation in HT regioregularity as a function of cobalt oxide concentration is shown in Figure 4 and decreases with increasing cobalt oxide content in the composites. The interaction between cobalt oxide and 3HT monomers may make the HT orientation less preferential.^49,52^

**Table 2:** Dyad and Triad Percentages for P3HT Polymer and P3HT/Co_3_O_4_ Composites

Sample	%HT	%HH	%HT–HT	%HT–HH	%TT–HT	%TT–HH
P3HT	84.45	15.55	70.74	10.61	9.65	9.00
P3HT/Co_3_O_4_ 3 wt.%	82.75	17.25	73.11	9.51	9.89	7.49
P3HT/Co_3_O_4_ 5 wt.%	82.64	17.36	73.89	9.98	8.89	7.23
P3HT/Co_3_O_4_ 8 wt.%	81.77	18.22	71.81	9.25	10.13	8.81

The dyad signals (Figure 5) broadened as the Co_3_O_4_ content increased. Additionally, at a high Co_3_O_4_ concentration (P3HT/Co_3_O_4_ 8 wt %), the HT dyad signal lacked the shoulders observed in P3HT. The above two observations are likely indicative of the strong interaction of P3HT with cobalt oxide.^21^ Similar broad signals have been reported for hydrogens in other materials, such as P3HT/MWNT,^53^ P3HT/[FeO­(OH)][Bibr ref21] systems, and P3HT/CdS composites.^54^ This broadening was attributed to interactions between the two materials in the composite and to the polymer’s aggregation around the inorganic component.

**Figure 5:**
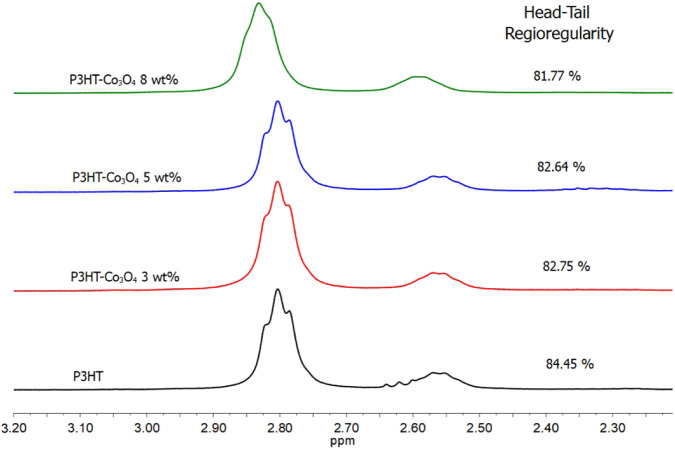
^1^H NMR spectrum of P3HT/Co_3_O_4_ composites showing differences in H-T regioregularities.

### 4.4 SEM (EDS), TEM, and AFM Analysis

SEM and TEM micrographs, together with EDS and AFM results, clearly show how the incorporation of cobalt oxide modifies the morphology of P3HT (Figure 6). The SEM image shows a rougher surface with higher-contrast areas, indicating the presence and dispersion of Co_3_O_4_ nanoparticles in the polymer matrix. TEM analysis revealed oxide nanoparticles with an average particle size of 23 nm. The particle size distribution was analyzed using ImageJ, and the corresponding histogram is shown in Figure 6b. The EDS spectrum shows characteristic peaks of Co and O, indicating their incorporation into the material. The AFM images show that the composite films at a concentration of 3 wt % exhibit surface morphology with a scarce presence of aggregates, whereas the 5 wt % composite shows more numerous and more prominent aggregates. The 8 wt % composite presents a higher number of aggregates, but with a more uniform distribution across the surface. This trend is consistent with P3HT nanocomposites in which, beyond a filler threshold, the system may transition from isolated domains to a more connected (percolation-like) micrometer-scale morphology. This trend is consistent with P3HT-based nanocomposites^55^ in which, once a filler loading threshold is exceeded, the system can transition from isolated domains to a more connected morphology (see Figure S5 for further details). A similar behavior was observed in Co–P3HT films, which is associated with electrostatic attraction between thiophene and Co^2+^, leading to increased P3HT conductivity via Co doping and significantly improving PSC performance. This topographic modification confirms the effective interaction between the polymer and the metal oxide, capable of altering the local organization of the P3HT chains and opening new charge-transfer pathways, which would enhance the conductivity of the 8 wt % composite over the 5 wt % composite. This is consistent with the lower photoluminescence observed for the 8 wt % composite, which exhibits stronger quenching than the 5 wt % composite, and may translate into more efficient hole extraction and transport when used as a hole-transport layer (HTL). Overall, the results confirm that the Co_3_O_4_ nanoparticles are successfully incorporated into the polymer network, promoting greater surface heterogeneity and an active interface between the two phases, conditions that can improve electronic mobility and ion diffusion.

**Figure 6:**
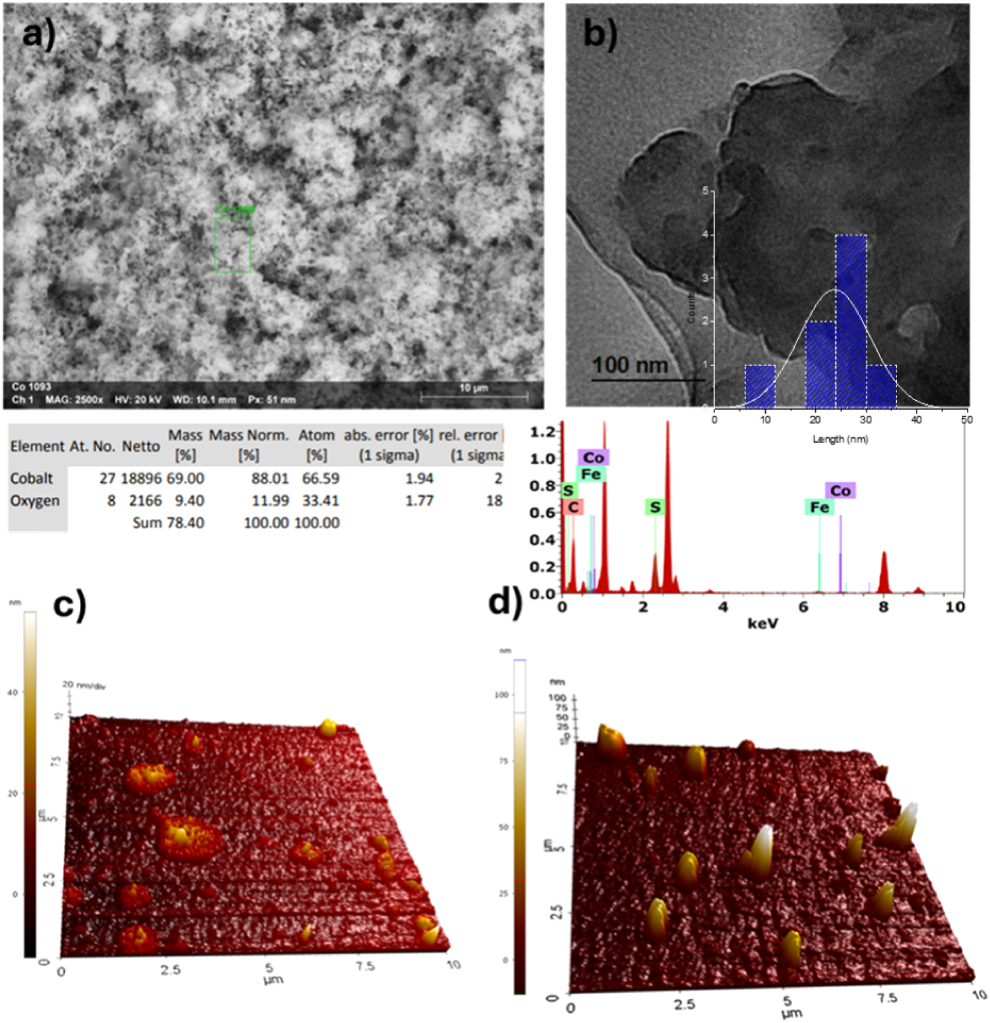
Analysis of (a) SEM-EDS P3HT and (b) TEM-EDS P3HT/Co_3_O_4_ (5 wt %), (c) AFM P3HT, (d) AFM P3HT/Co_3_O_4_ (5 wt %).

### 4.5 Adsorption of P3HT on Co_3_O_4_(110): Geometry and Energy

Before surface modeling, we noted that in the Co_3_O_4_ bulk structure (Figure 7a), two types of Co atoms are present: tetrahedral (Co_tet_) and octahedral (Co_oct_). There are two possible terminations for the Co_3_O_4_(110) surface: oxygen-poor (typically known as A) and oxygen-rich (B).^27,29^ For this study, we modeled B structure (Figure 7b) based on the results of Selcuk and Selloni, (2015),^27^ who concluded that surface B is exposed under ambient conditions. To define the most probable binding sites and orientation of the polymer molecule, charge density maps colored by the electrostatic potential of the polymer and the surface of interest were obtained (Figures S2 and S3 respectively), this way the thioether moiety showed a main density accumulation with a negative electrostatic potential, nonetheless the thiophene ring presented a negative electrostatic potential accumulation around the ring area. On the other hand, in the Co_3_O_4_(110) surface, the topmost Co_oct_ atoms showed positive regions, and the oxygen atoms present a negative electrostatic potential. This gave us a clue that the surface Co_oct_ atoms may be the most likely to bind sites in the Co_3_O_4_(110) surface and that the P3HT structure (Figure 7c) could interact with them by the thioether moiety or the thiophene ring.

**Figure 7:**
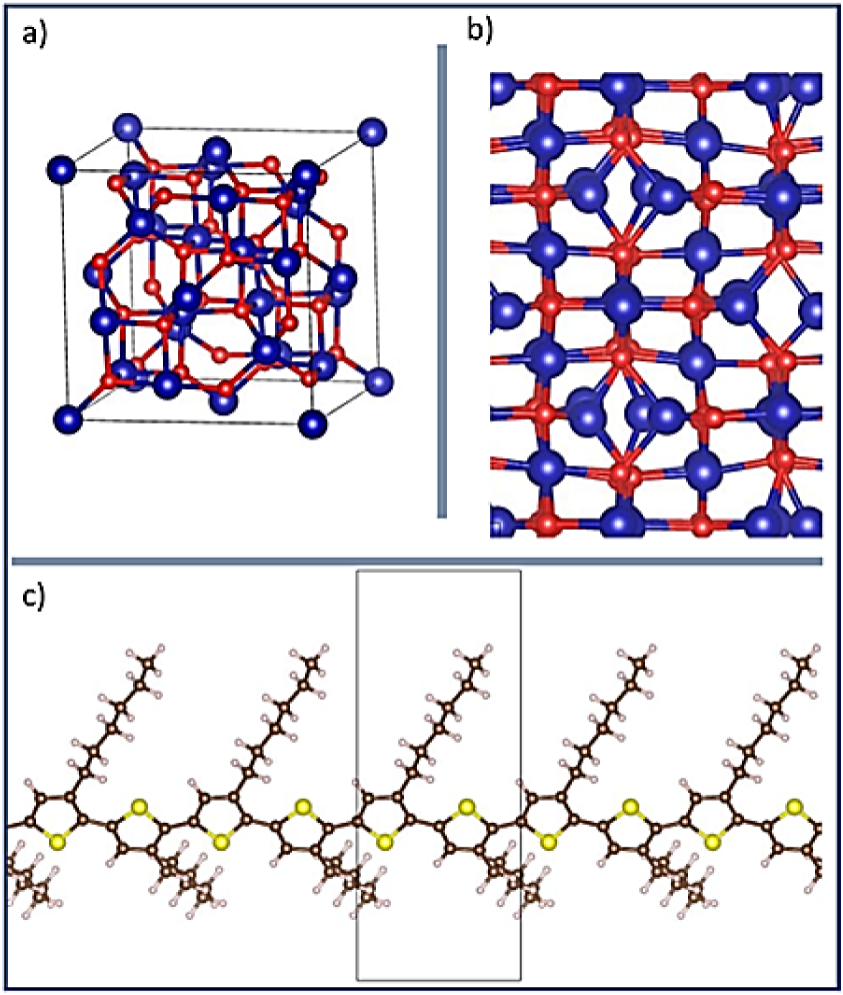
Models employed in this study. (a) Bulk Co_3_O_4_, (b) optimized Co_3_O_4_(110) surface, and (c) P3HT optimized geometry. Atom color code: Co (Blue), O (red), C (brown), H (white), S (yellow).

Different adsorption configurations were investigated. Figure 8 shows relaxed geometries of the most stable adsorption configurations of P3HT on the Co_3_O_4_(110) surface. The first configuration shown in Figure 8a was initially arranged, expecting an interaction between Co_oct_ and the thiophene ring; however, the structure relaxed, forming coordination bonds between S and the Co_oct_ atoms. The angle formed between this bond with respect to the surface was 80.36°. In this configuration, the thiophene rings relaxed near the surface Co atoms; the Co–S bond distance was 2.22 and 2.23 Å, respectively. The calculated adsorption energy for this model was −3.21 eV. This value is close to the value reported by Özçelik and coworkers,^56^ who studied the adsorption of P3HT on different metallic substrates (Ag, Au, and Pt) using DFT methods, finding binding energies of 2.92, 2.88, and 2.11 for each metal, respectively. The relaxed structure presented in Figure 8b resulted in a similar comparison to the previous model; the main difference is the tilt angle between each thiophene moiety, which resulted in a slightly more tilted configuration relative to each other than Figure 8a, and hence the carbon ring atoms are more distant from the surface Co atoms. This model also showed coordination bonds between the Co_oct_ and S atoms, resulting in a slightly lower adsorption energy (−3.15 eV). This difference indicates that van der Waals interactions can make an important contribution to the adsorption energy. These results are concordant with the observed shift in the FT-IR band at 820 cm^–1^, implying that the reduced vibratory freedom is indeed caused by the interaction between the oxide surface and P3HT molecule, while this rigidity comes mainly from the Co–S bond formation, π-type and van der Waals attractions could also have an important contribution.

**Figure 8:**
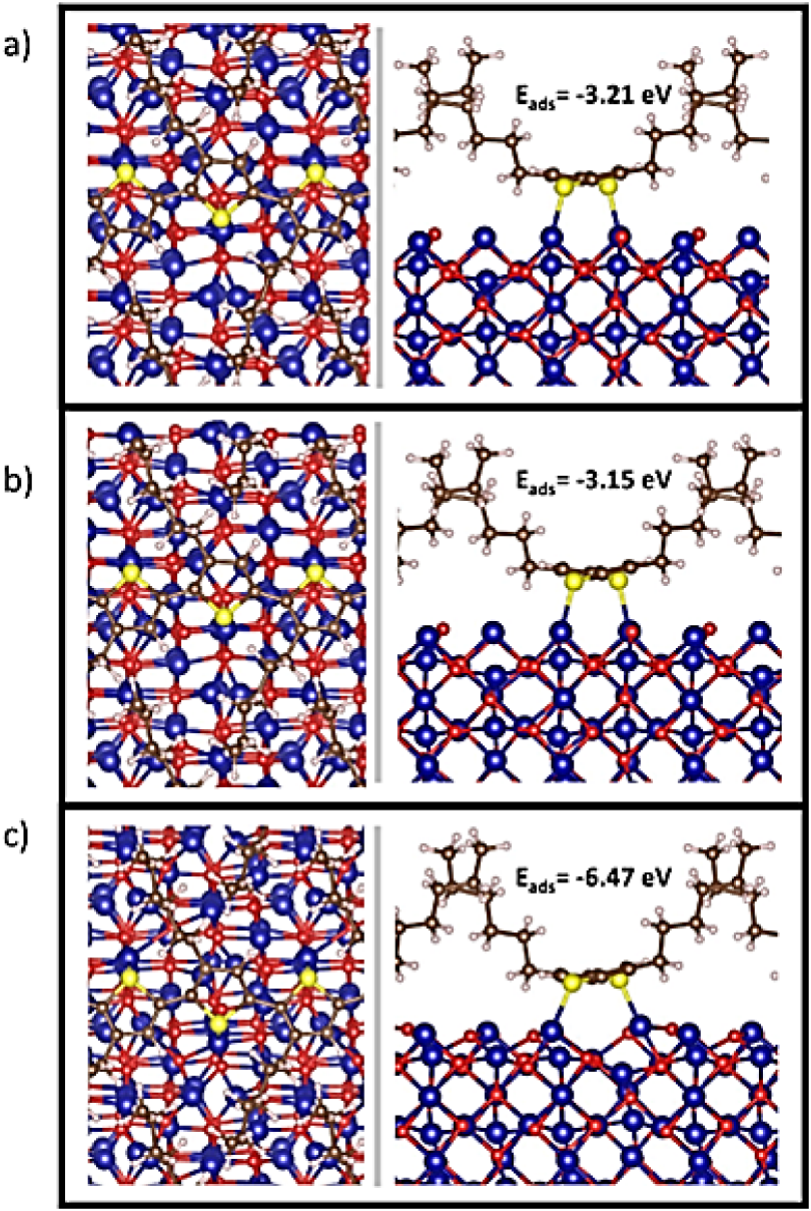
Adsorption models of P3HT on the Co_3_O_4_(110) surface. (a) Thiophene is almost flat. (b) Thiophene is slightly tilted. (c) Thiophene is bound square planar, distorted.

The third relaxed structure (Figure 8c) showed an important difference in geometry compared to the other two. In this model, the thioether function formed a coordination bond with a Co_oct_ atom; however, the angle between this bond and the surface was smaller than in the previous two models (72.62°). This value was closer to the bulk configuration of the Co_oct_. On the other hand, one of the two Co_oct_ atoms adopted a coordination number of four instead of five, adopting a distorted square-planar geometry, which represents a change in the surface geometry. Furthermore, the adsorption energy was −6.47 eV, twice that of the two previous models. If we compare this result with that reported by Özçelik and coworkers,^56^ it seems to be almost double; however, in their model, they show that only one sulfur is close enough to the metal to form a coordination bond and argue that, for each S-metal bond, the reported bond energies are around 2 eV; considering that explanation, our result seems reasonable. On the other hand, the smaller angle of the Co–S bond to the surface-reducing group reduces the distance between the molecule and the surface, thereby plausibly improving vdW interactions. Interestingly, all three adsorption models tended to chemisorb via Co–S bonds rather than coordinate via π-Co interactions. To better understand these behaviors, a study of charge transfer and noncovalent interactions was conducted; the results are presented in the next section.

#### 4.5.1 Charge Transfer and Noncovalent Interactions

To have a deeper insight into the character of the bonds formed between the polymer and the surface (Co_3_O_4_), charge transfer was quantified using the quantum theory of atoms in molecules QTAIM.^35^ The results of Bader’s charge analysis are summarized in Table 3. We observed that the net charge for each cobalt atom bond is positive, with values ranging from +1.26 to +1.56 e. These results were like those reported by Selcuk and coworkers^27^ who showed values slightly lower than 1.5 for Cooct on the B-type (110) surface. The sulfur atoms bound to the protein showed a net charge of +0.11 to +0.15 e. These results imply that the bonding is covalent with ligand-to-metal charge transfer.

**Table 3:** Bader Charge Analysis for S and Co Atoms for Each Modeled Configuration

Configuration	Bond	Atom	Net charge
a	Co–S	S	+0.15
		Co	+1.30
	Co–S	S	+0.13
		Co	+1.30
b	Co–S	S	+0.14
		Co	+1.28
	Co–S	S	+0.11
		Co	+1.28
c	Co–S	S	+0.14
		Co	+1.56
	Co–S	S	+0.14
		Co	+1.26

On the other hand, the NCI index helped to identify the long-range interaction contribution to adsorption. The NCI mapping, represented as the density-colored RDG isosurface (isovalue = 0.5), and the RDG vs ρsign­(λ_2_) plot are shown in Figure 9 for the adsorption models of P3HT on Co_3_O_4_. The red, green, and blue colors depict repulsion, van der Waals interactions, and attraction, respectively. Figure 9a shows the “flat thiophene ring” configuration revealing the coordination bonds formed between Co and S as a blue solid ring appearing at −0.10 density value, attractive van der Waals interactions, a large green isosurface region around the thiophene rings observed in the RDG plot at density values of −0.011, a slightly yellow component appeared between the C atoms of the thiophene moiety chain and the surface oxygen atoms, corresponding to weak repulsions observed in the RDG plot at density values of +0.011, finally, a small red repulsion component between sulfur and nearest oxygen atoms appearing in the RDG plot at density values of +0.075. The model results in Figure 9b are very similar to those of the previous one; however, the weak repulsion component appears to cover a larger area, corresponding to the repulsion between oxygen and the delocalized doubly bonded C atoms in thiophene. This larger repulsion component could be responsible for the slightly less negative adsorption energy observed in this model compared to that in Figure 9a. On the other hand, the coordination bond also appeared as an intense blue ring, and van der Waals attractions were clearly observed between the Co atoms and the delocalized doubly bonded C atoms. Finally, Figure 9c shows the NCI isosurface map and the RDG plot for the configuration with a lower Co–S bond angle with respect to the surface. A large van der Waals attraction region was observed, concentrated mainly on the thiophene ring near the surface Co atoms, with a density of −0.016. The Co–S coordination bonds were observed as intense blue donuts; however, in this case, one of them showed a small contribution to the red. This was observed in the RDG plot at −0.09 and +0.09 of the density value for the attraction and repulsion, respectively. It is evident that van der Waals attractions play an important role in the highly negative value of the observed adsorption energy; on the other hand, the difference in the coordination number of one of the Co atoms bound to the thioether moiety, as well as the smaller bond angle with respect to the surface, apparently also have an important contribution to the observed adsorption energy. The type of interaction driving the P3HT adsorption on the Co_3_O_4_ surface becomes clearer when the surface adsorption simulation results are compared to the ^1^H NMR results (Figure 5) where the degree of HT regioregularity diminishes as the oxide concentration increases, indicating that a less sterically hindered structure is more likely to produce stronger Co–S bonds and less van der Waals repulsions, supporting that Co–S bonds drive the adsorption process.

**Figure 9:**
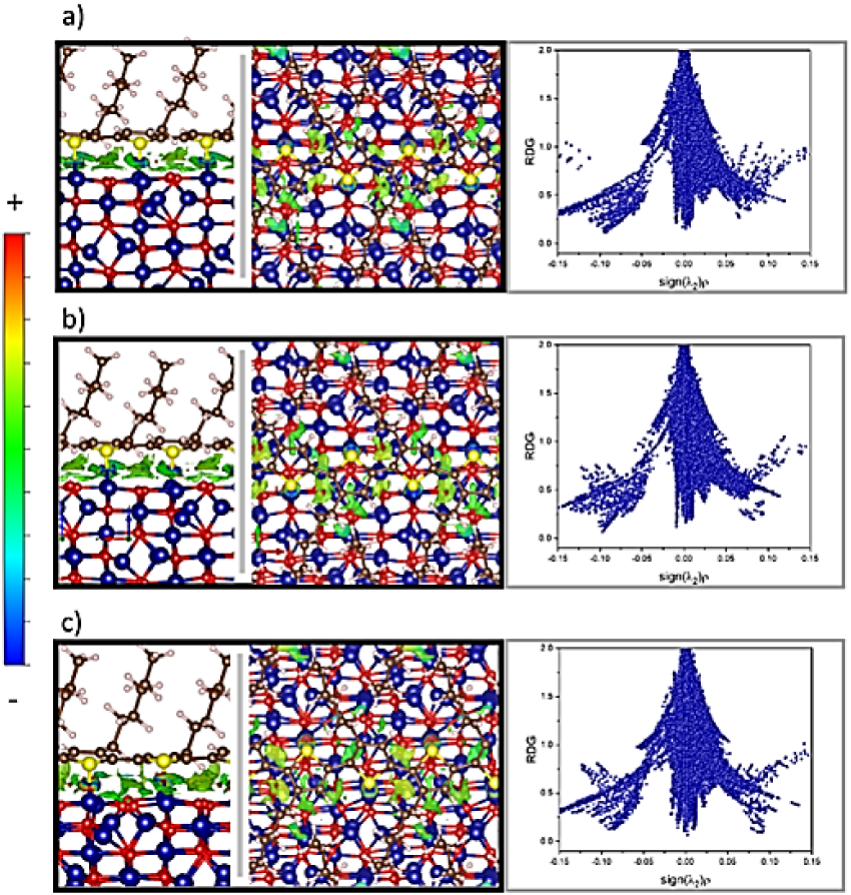
Noncovalent interactions are represented as the isosurfaces of reduced gradient colored by the density values (top and side view, isovalue = 0.5), and the plot of the reduced gradient vs the density times the sign of the second eigenvalue of the Hessian matrix for the different adsorption configurations (a, b and c respectively). Color scale indicates sign­(λ2)­ρ value, with blue as highly negative and red as highly positive. Strong attractive interactions are shown in blue, van der Waals attractions in green, and steric hindrance repulsions in red.

## 5 Conclusions

The experimental and theoretical study provides insight into the interaction between P3HT and Co_3_O_4_ in thin hybrid films. In situ oxidative polymerization enabled the integration of both components, yielding homogeneous nanocomposites in which the oxide serves not only as an inorganic filler but also as an active electronic component that modifies the polymer’s structural and optoelectronic properties. Spectroscopic and microscopic results indicate that Co_3_O_4_ nanoparticles interact directly with the P3HT backbone. FTIR-ATR spectra revealed subtle but significant band shifts, especially at 820 and 1115 cm^–1^, indicating sulfur–cobalt coordination. This observation was complemented by photoluminescence measurements, in which the appearance of a sharper, slightly blue-shifted emission band confirmed molecular rearrangement and a partial modification of the conjugation length, indicating enhanced P3HT conductivity and positioning it as a candidate for use as a hole-transport layer (HTL) in solar cells. The ^1^H NMR analysis also showed a reduction in HT regioregularity with increasing oxide content, suggesting that the oxide interferes with the orientation of polymer growth during polymerization. Morphological studies (SEM, TEM, AFM) confirmed a well-defined dispersion of the Co_3_O_4_ lamellar structures and an increase in surface roughness, suggesting the formation of heterogeneous domains favorable for interfacial charge transport. DFT+U calculations strongly support these findings. Theoretical modeling of P3HT adsorption on the Co_3_O_4_(110) surface demonstrated that sulfur atoms preferentially coordinate to the cobalt octahedral centers via S–Co covalent bonds, accompanied by ligand-to-metal charge transfer and significant van der Waals contributions. These interactions stabilize the polymer-oxide interface and explain the experimentally observed electronic coupling. This synergy between organic and inorganic components enables the design of high-performance hole-transporting layers or photoactive hybrids with tailored energy alignment.

## Supplementary Material



## Notes

Characterization data, including TGA thermograms; charge density isosurfaces colored by electrostatic potential for Co_3_O_4_ and P3HT; computational parameter optimization for bulk Co_3_O_4_; SEM image histogram (PDF)
