# Supplementary material for: Electronic-Structure Role of Cobalt Oxide Nanoparticles in P3HT-Based Composites: From Thin-Film Fabrication to DFT-Assisted Surface Interaction Analysis
Source: ACS Omega. 2026 Apr 22;11(17):25683–92. doi: 10.1021/acsomega.6c00453 (PMC13150650; doi:10.1021/acsomega.6c00453)
Supplement: Supplementary file 1 [file ao6c00453_si_001.pdf]

Electronic-Structure role of cobalt oxide nanoparticles in P3HT-based composites: from thin film fabrication to DFT-assisted surface interaction analysis

Diego Hernández-Martínez [a], Luis E. López-González [b], Sarahi García-Carvajal [c]*, Aurora M. Pat-Espadas[a], Karla A. López-Gastelum [d], Damian F. Plascencia-Martínez [e], Guillermo Suárez-Campos [f], María E. Nicho-Díaz [g]*

[a]Departamento de Ingeniería Química y Metalurgia, Universidad de Sonora, Del Conocimiento, Centro, C.P. 83000 Hermosillo, Sonora, México
[b]Centro de Nanociencias y Nanotecnología, Universidad Nacional Autónoma de México Km 107 Carretera Tijuana-Ensenada s/n, Ensenada, B.C., C.P. 22800, México
[c]Escuela Nacional de Estudios Superiores Unidad León, Universidad Nacional Autónoma de México, Boulevard UNAM #2011 Col. Predio El Saucillo y El Potrero Comunidad de Los Tepetates, C.P. 37684, León, Guanajuato, México
[d]Departamento de Investigación y Posgrado en Alimentos, Universidad de Sonora, Rosales y Blvd. Luis Encinas, C.P. 83000 Hermosillo, Sonora, México
[e]Coordinación de Tecnología de Alimentos de Origen Vegetal, Centro de Investigación en Alimentación y Desarrollo, A.C., Carr. Gustavo E. Astiazarán Rosas No. 46, Col. La Victoria, C.P. 83304, Hermosillo, Sonora, Mexico
[f]Departamento de Investigación en Física, Universidad de Sonora, Blvd. Luis Encinas y Rosales S/N, Hermosillo, Sonora 83000, México
[g]Centro de Investigación en Ingeniería y Ciencias Aplicadas, Universidad Autónoma del Estado de Morelos, Av. Universidad 1001, Col. Chamilpa, C.P. 62209 Cuernavaca, Morelos, México
** E-mail:diego.hernandez@unison.mx, scarvajalg@enes.unam.mx.*

Supporting Information

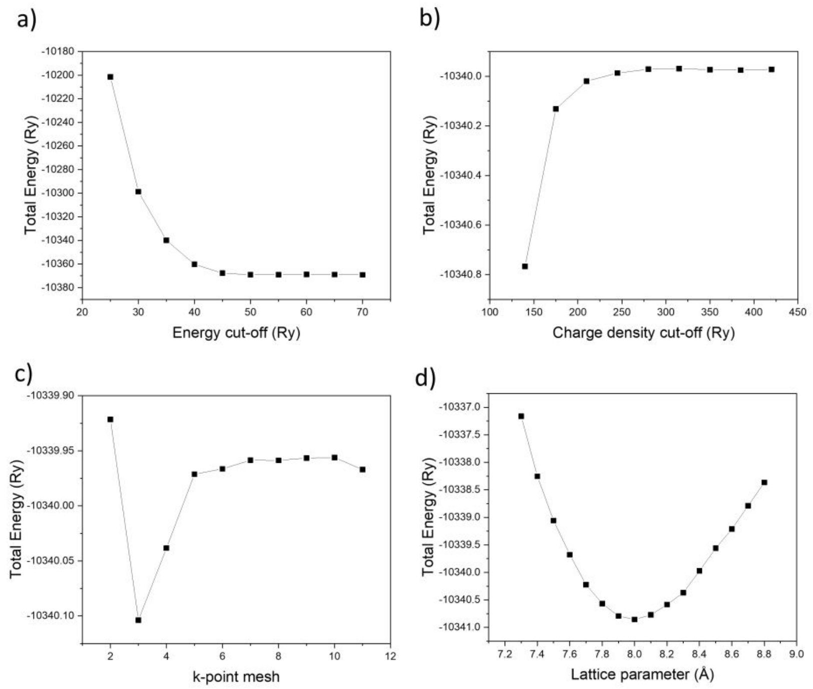

Figure S1. Optimization of computational parameters for bulk Co_3O_4. The total energy of the system as a function of (a) plain wave energy cut-off, (b) charge density energy cut-off, (c) k-points, and (d) lattice parameter.

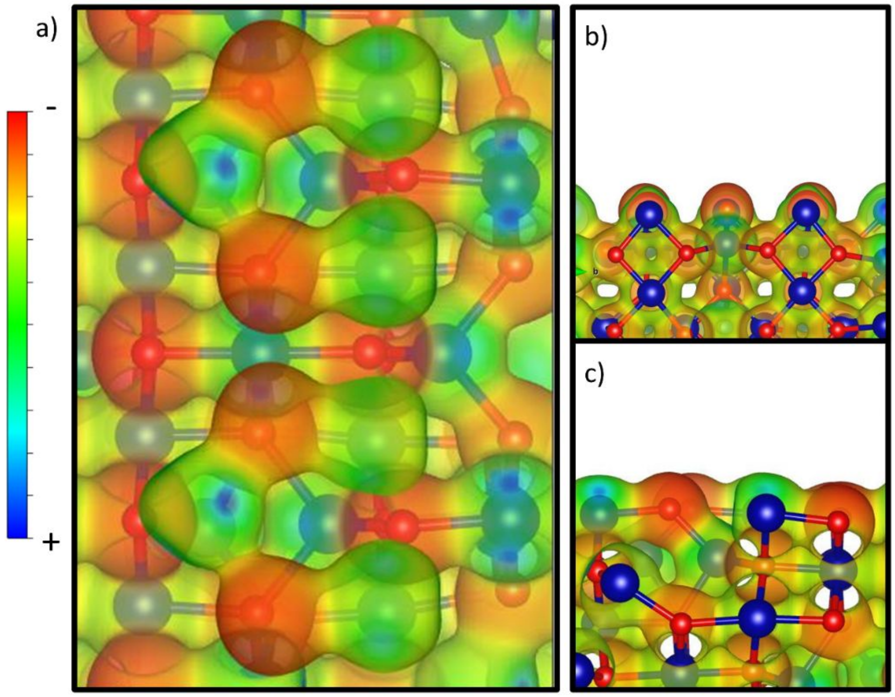

Figure S2. Charge density isosurfaces colored by the electrostatic potential for Co_3O_4 (110) surface as seen from (a) top, and (b and c) lateral views. The color scale indicates a positive electrostatic potential as blue and a negative one as red. Atom color code: Co (blue), O (red).

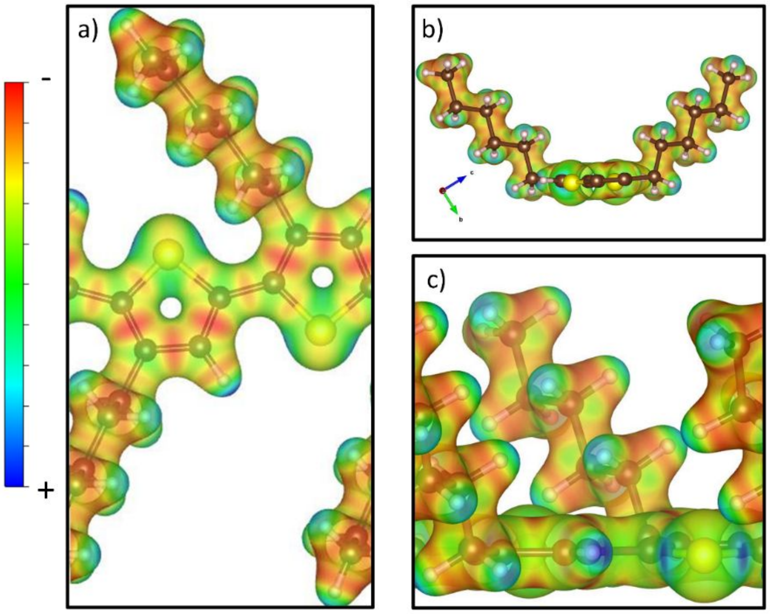

Figure S3. Charge density isosurfaces colored by the electrostatic potential for P3HT as seen from (a) top, and (b and c) lateral views. The color scale indicates a positive electrostatic potential in blue and a negative one in red. Atom color code: S (yellow), O (red), C (brown), H (white).

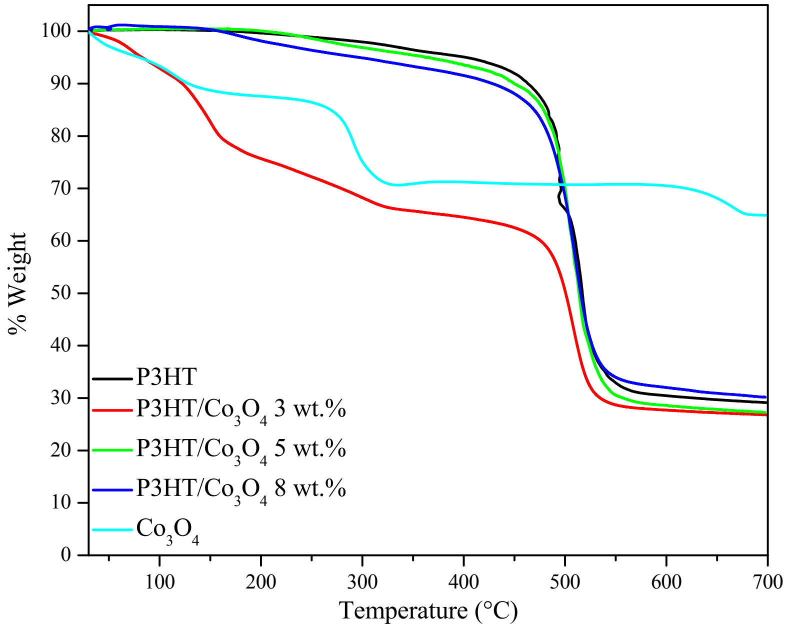

Figure S4. TGA of P3HT, Co_3O_4, and their composites at different concentrations.

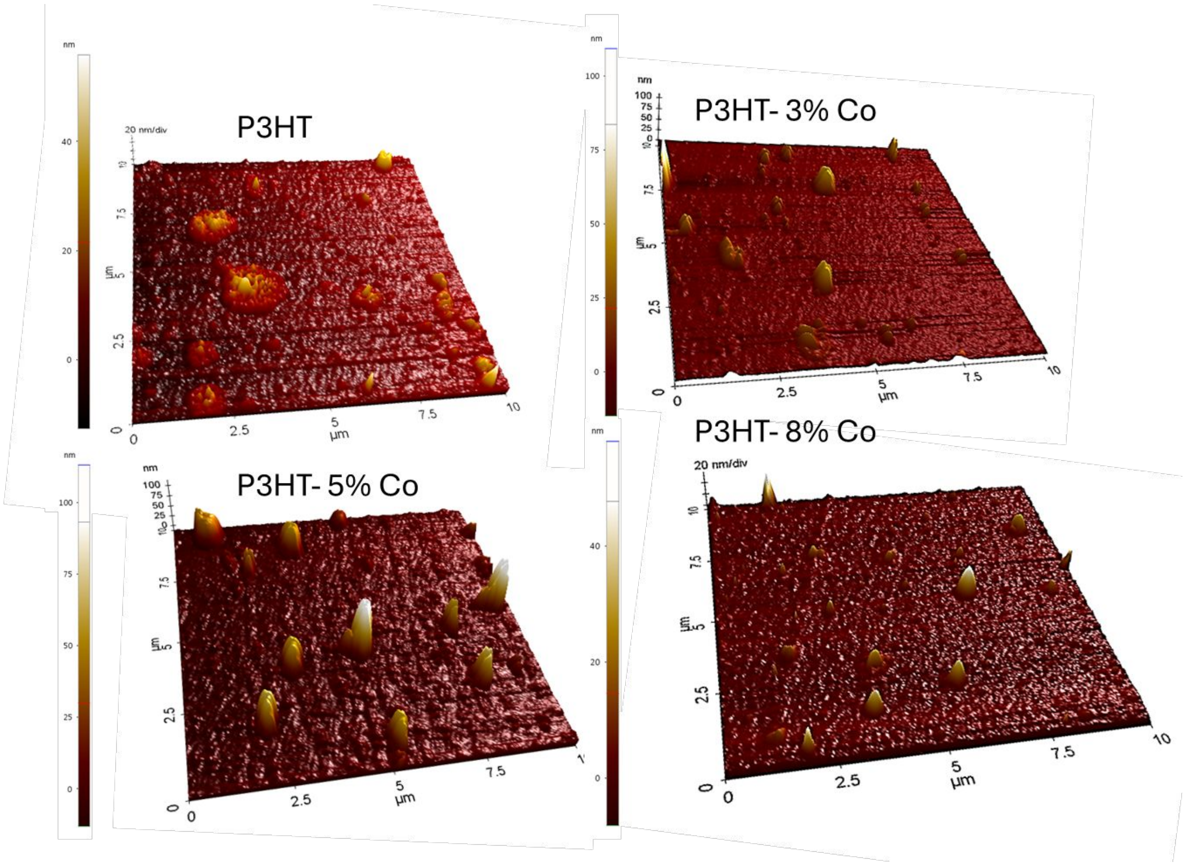

Figure S5. AFM Analysis of P3HT, P3HT/Co_3O_4 3%, 5% and 8% wt.%.

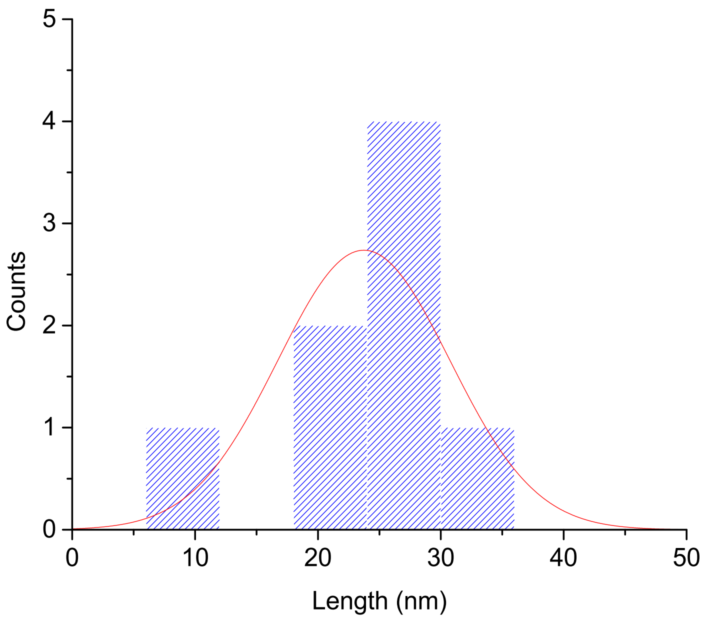

Figure S6. Histogram of particle size distribution of Co_3O_4.
